# The Diabetes Eating Problem Survey-Revised (DEPS-R) in a Greek Adult Population with Type 1 Diabetes Mellitus: Model Comparison Supporting a Single Factor Structure

**DOI:** 10.3390/nu13072375

**Published:** 2021-07-12

**Authors:** Calliope Karastogiannidou, Parthena Giannoulaki, Ioannis Samaras, Evangelia Kotzakioulafi, Triantafyllos Didangelos, Ioana Corina Bocsan, Emilia Vassilopoulou

**Affiliations:** 1 Department of Nutritional Sciences and Dietetics, International Hellenic University, 57400 Thessaloniki, Greece; karasto@ihu.gr (C.K.); samarasgiannis15@gmail.com (I.S.); vassilopoulouemilia@gmail.com (E.V.); 2 Diabetes Center, 1st Propaedeutic Department of Internal Medicine, Medical School, University General Hospital of Thessaloniki AHEPA, Aristotle University of Thessaloniki, 54636 Thessaloniki, Greece; nenagian@yahoo.com (P.G.); ekotzakioulafi@gmail.com (E.K.); didang@auth.gr (T.D.); 3 Department of Pharmacology, Toxicology and Clinical Pharmacology, “Iuliu Hatieganu” University of Medicine and Pharmacy, 400337 Cluj Napoca, Romania

**Keywords:** DEPS-R, Greek validation, confirmatory factor analysis, model comparison, type 1 diabetes mellitus, disordered eating, diabulimia

## Abstract

Type 1 diabetes mellitus (T1DM) patients occasionally develop disordered eating behaviors, leading to insulin manipulation without medical consultation, targeting to achieve weight control. In clinical practice, the Diabetes Eating Problem Survey-Revised Version (DEPS-R) questionnaire has been used to evaluate eating disorders in T1DM patients. This study was conducted to validate the factor structure of the Greek version of DEPS-R using Confirmatory Factor Analysis (CFA), to investigate its reliability and convergent validity in Greek T1DM adults and to compare a single factor DEPS-R model with multiple factor models. Participants were 103 T1DM adults receiving insulin, who responded to DEPS-R. Their anthropometric, biochemical and clinical history data were evaluated. The sample presented good glycemic control and 30.1% scored above the established DEPS-R cut-off score for disturbed eating behavior. CFA results revealed that the data fit well to the factor models. The DEPS-R scale had good reliability and was positively linked to BMI, HbA1c, total daily dose and time in range. Model comparison supported the superiority of the 1-factor model, implying that Greek clinicians and practitioners might not have to consider individualized treatment based on various scores across different subscales but they can adopt a single DEPS-R score for an easy and efficient screening for disordered eating.

## 1. Introduction

### 1.1. Diabetes Mellitus and Diabulimia

Diabulimia is a term, derived from diabetes mellitus (DM) and bulimia, for an eating disorder (ED) characterized by the intentional reduction or omission of a dose of insulin without consulting a doctor, in order to control weight [1]. Individuals with type 1 DM (T1DM) may do this hoping to lose weight, because, as insulin is an anabolic hormone that helps with weight gain, following high carbohydrate intake, any excess carbohydrate is converted to fat, leading to weight gain [2]. By reducing the prescribed insulin dosage, the person with T1DM tries to achieve weight loss through acidosis, which causes urination and elimination of excess glucose (glucosuria). This process drives the body to burn fat to counteract the lack of available energy, which leads to the release of ketones, resulting in ketoacidosis [3,4].

Diabulimia can be classified as an ED and as a behavioral disorder, but it may also be associated with fear of hypoglycemia, lack of medication because of inadequate medical coverage, or an overall poor psychological state. Regardless of the underlying reasons, withholding insulin results in ketoacidosis, a condition that causes various complications in the body, especially when persisting for a long time [5].

Patients with diabulimia have particular eating patterns. On the one hand, they avoid sweets and fats, limit their food intake and skip meals in order to lose weight; on the other hand, they may consume large amounts of food and feel guilty, and, in the end, they reduce or omit their insulin dosage, as described above, for purposes of “purification” [3].

### 1.2. Factors Associated with Development of EDs in People with T1DM

One of the main factors leading people with DM to EDs is their body image. The negative emotions they experience when they observe their body may culminate in depression, anxiety and other mental illnesses, but may also trigger outbursts of overeating or binge eating or concerted efforts to reduce their weight [2]. Other factors may be negative comments from family, friends and acquaintances [2,6], and possibly declined sex life and doubts about their acceptance by the opposite sex. Another, equally important, factor is that people with DM must constantly practice carbohydrate counting, which can lead to intensive examination of what they consume, and may culminate in extreme ED, resulting in blocking of macronutrients or whole categories of foods [2,3,7].

### 1.3. The Diabetes Eating Problem Survey-Revised Version (DEPS-R)

Many screening tools are available to identify people who are at risk of developing EDs or already present an eating problem, but only one screening tool has been validated for ED in T1DM, the Diabetes Eating Problem Survey-Revised Version (DEPS-R) [8,9,10]. This tool has recently been reported as the best validated tool for adolescents and adults [11], and is widely used to study people with T1DM who are at risk of developing or have already developed diabulimia. An increased risk is indicated by scores of >20 [8].

This was the first screening tool for EDs designed specifically for patients with T1DM, and its psychometric properties among children and adolescents have been validated in German [12], French [13], Spanish [14], Turkish [15], Italian [9] Norwegian [10,16], and Chinese [17]. The Italian version of the DEPS-R provided initial support for the validity and reliability of the instrument [9], and the Norwegian version showed adequate psychometric properties when used with adults [10]. Apergi and colleagues (2020) reported that the Greek version of the DEPS-R is a valid, self-administered T1DM-specific screening tool for adults [18].

The DEPS-R was initially developed as a single factor instrument [8], but later research supported division into three factors, named “eating habits”, “thinness” and “high blood glucose” [9,10,16,17], or four [13] or even more factors, i.e., the aforementioned three factors plus at least one other factor, named “avoidance” [14]. This has important implications in practice, because while a single scale score is used for easy screening for disordered eating, a multi-scale DEPS-R requires individualized treatment based on the different responses of patients across the multiple scales of the tool [10], which complicates matters for clinicians and therapists. Only one study, to date, has reported findings from both a single factor model and multiple factor models [10], but, even in that study, the models were not nested and therefore they were not directly comparable, as the authors recognized.

### 1.4. Objective of the Present Study

The present study aims: (a) to validate the factor structure of the Greek version of DEPS-R using confirmatory factor analysis (CFA), which was not reported in the initial Greek study; (b) to investigate the reliability and convergent validity of the Greek version of the DEPS-R in a sample different from that of Apergi and colleagues (2020) [18]; and (c) to compare a single factor DEPS-R model with multiple factor models.

## 2. Materials and Methods

### 2.1. Participants

The present study was conducted with patients aged > 18 years with a diagnosis of T1DM, who had a good knowledge of the Greek language. The exclusion criterion was the diagnosis of T1DM of less than one year. The initial goal of the study was to involve at least 100 patients with T1DM.

The study protocol and questionnaire were approved by the University General Hospital AHEPA (protocol code 33069) and all the procedures followed were in accordance with the Declaration of Helsinki (1964) and GDPR.

### 2.2. Screening Tool

The Greek version of DEPS-R [18] was administered to identify EDs and unhealthy weight loss practices in the study population in the 4 weeks before the completion of the questionnaire. The questionnaire consists of 16 items, and the responses are recorded on a 6-point Likert scale, ranging from 0 = never to 5 = always, where the higher the score of each patient, the greater the risk of ED. An increased risk is indicated by scores of >20.

Based on their scores on DEPS-R, the participants were divided into two categories, those with a normal score (<20) and those with a high score (>20).

### 2.3. Sample and Data Collection Procedure

All 142 patients who attended the T1DM outpatient clinic of the AHEPA hospital in Thessaloniki between 17 September 2020 and 17 December 2020 were approached after an appointment with their doctor. Of these, 103 adult patients with T1DM, the majority of whom were women (75.7%), were included in the study.

The participants provided their consent to participate in the study before completing the questionnaire. They were informed of the possibility to withdraw from the study whenever they wished and could request clarification when completing the questionnaire. They gave permission for review of their medical records to supplement clinical data. All responses were treated confidentially. Each patient had a code number in order to ensure the protection of sensitive personal data. This number was recorded with the data we collected.

A dietitian completed the questionnaires in face-to-face appointments to reduce contact via documents, strictly following COVID-19 personal protection measures. However, the outbreak of cases with coronavirus in early November 2020 forced the Greek Government to stop all outpatient clinics’ operations, after which the questionnaires were fulfilled via Zoom and Skype meetings. Τhe 16 questions of DEPS-R were asked one by one from the respondents with the necessary clarifications when requested. Τhe average time taken to complete the questionnaire was 12 min.

Clinical, anthropometric and biochemical data were retrieved from the medical records. Body mass index (BMI) (kg/m^2^) was calculated from weight (kg) and height (m). Clinical history data included: type of insulin therapy in multiple daily injections (MDI) or continuous subcutaneous insulin infusion (CSII), with or without continuous glucose monitoring (CGM), total daily dose (TDD) and frequency of blood glucose monitoring (from patient calendar data or data from glucose meters or CGM-14 day), frequency of hypoglycemia (from log calendars or from CGM-14 day), time in range (TIR-14 day from CGM), time above range (TAR 14 day from CGM) > 180 mg/dL and time below range (TBR 14 day from CGM) < 70 mg/dL, which were answered either approximately by the patient or from data in the insulin pump system or the continuous glucose recording system. The biochemical data recorded were fasting blood glucose (FBG) (mg/dL) and glycosylated hemoglobin (HbA1c) (%), from memory recall of the last measurements or from the records.

### 2.4. Analytical Approach-Statistical Analysis

Statistical analysis was conducted using the SPSS v.24 statistical package and p-level was set at 0.05. Normality of distribution was assessed both visually and through the Kolmogorov–Smirnov test. Quantitative variables were expressed as mean values (SD) or as median values (interquartile range = IQR) and categorical variables using absolute and relative frequencies. For comparison between groups, the independent samples *t*-test was performed. When normality of distribution criterion was not fulfilled, the Mann–Whitney U test was employed. Pearson’s coefficient was used to assess correlation between continuous variables. When normality of distribution criterion was not fulfilled, Spearman’s rho was used. Logistic regression analysis was used to identify predictors of a positive DEPS-R screen. Variables that exerted a statistically significant association univariately were entered into a multiple logistic regression model and subsequently removed at *p* > 0.10 in a backward elimination strategy. Odds Ratios (OR) are presented along with their 95% Confidence Intervals (CI). The same procedure was followed for the total score of DEPS-R subscales. All variables found to be univariately associated with each DEPS-R subscale were entered into a multiple linear regression model and subsequently removed at *p* > 0.10 in a backward elimination strategy. Unstandardized beta coefficients are presented along with their standard errors. Internal consistency and reliability of the questionnaire was examined using Cronbach’s alpha.

The factorial structure of the Greek version of DEPS-R was examined using confirmatory factor analysis (CFA) with maximum likelihood estimation method [19,20,21]. Four nested models with one [8], two, three [9,10,17] and four factors were tested. The four-factor model contained the factors (eating habits/food attitudes, high blood glucose/bulimic behaviors, thinness/weight control, avoidance) that emerged in the Spanish study [14], but their fifth factor (restriction) with a single item was deemed inappropriate for CFA; thus, this item was linked with the “eating habits” factor, in line with factor analyses conducted in other countries [9,10,17]. The following fit indicators are presented in the results section: chi-square statistic with the respective *p*-value, comparative fit index (CFI), Tucker Lewis index (TLI), which is assumed to be less affected by sample size [22] and root mean square error of approximation (RMSEA). CFA was performed using the AMOS program, version 22. In line with an earlier study with Greek adults [18], item number 8 (I make myself vomit) had almost zero variance, which did not allow computation of covariance matrices and fit indices. Item 8 was also highly skewed to zero, implying that its exclusion would not affect the DEPS-R total score; hence, this item was not included in the CFAs. As in previous studies [10], modification indices suggested that errors of indicators of the “eating habits” factor should be allowed to covary. Accordingly, three correlated residuals were added, which remained the same across all models, allowing comparison between nested models. Although the rationale for correlated residuals in model 4 might be questionable because they stem from indicators belonging to different factors, the findings from model 4 are presented for illustrative purposes.

## 3. Results

### 3.1. Participants

The characteristics of the 103 patients participating in the study are presented in Table 1. The patients had a median age of 37 years and most were women (75.7%) and had normal BMI levels (Mean = 23.96). They had DM for approximately 18.3 years, the majority being treated by MDI insulin regimen (69.9%) and CGM (73.5%) use. Their glycemic control was relatively good (Median HbA1c = 6.80%).

In general, the patients presented good glycemic control, according to their FBG and HbA1c, and there was no difference between men and women. Although the women had borderline significantly lower TIR compared with the men (Median = 68.5 vs. 79.5, *p* = 0.058), the TAR, TBR and hypoglycemic events showed no difference between the two groups. The women, however, measured their blood glucose levels more frequently than the men (median = 7.0 vs. 5.5, respectively, *p* = 0.010). (Table 1).

### 3.2. DEPS-R Scale

**Figure 1:**
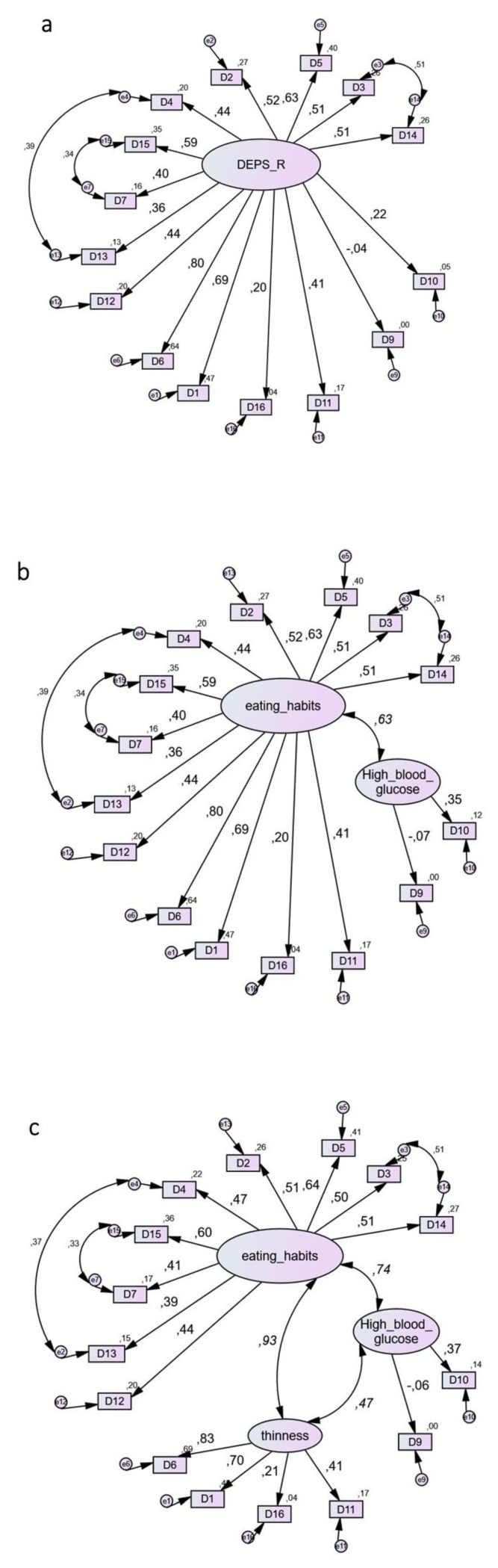

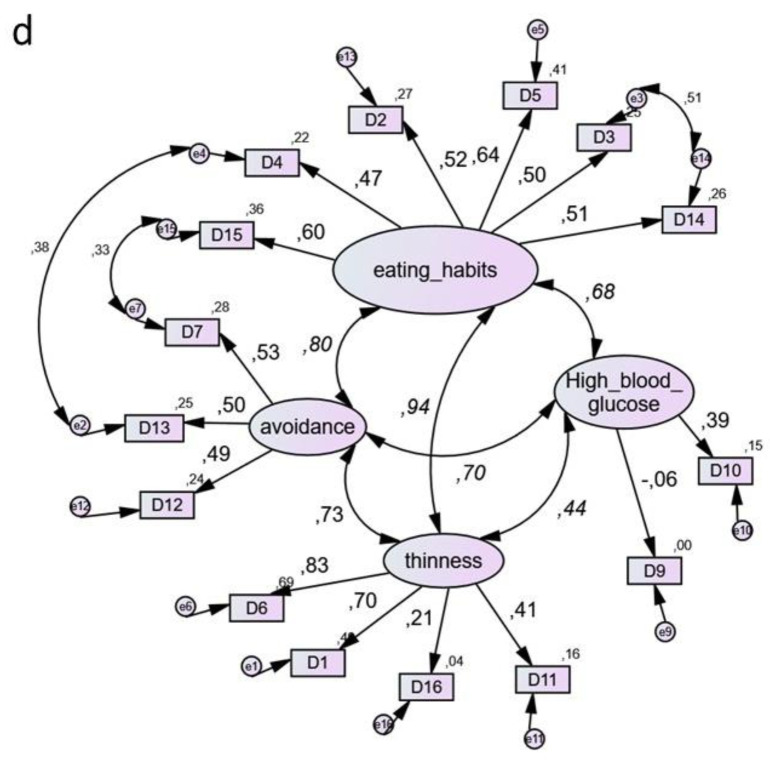
Use of the Diabetes Eating Problem Survey-Revised (DEPS-R) in a Greek adult population with type 1 diabetes mellitus: comparison between four-factor models. (**a**) Model 1. (The most parsimonious) Factors: (1) DEPS-R one single factor. χ^2^ = 102.1, df = 87, χ^2^/df = 1.17, CFI = 0.954, TLI = 0.944, RMSEA = 0.041, AIC = 168, ECVI = 1.65, ECVI LO-HI 90 = 1.53–1.97. (**b**) Model 2. Factors: (1) Eating Habits, (2) High Blood Glucose. χ^2^ = 102.1, df = 86, χ^2^/df = 1.18, CFI = 0.951, TLI = 0.940, RMSEA = 0.043, AIC = 170, ECVI = 1.67, ECVI LO-HI 90 = 1.51–1.96. (**c**) Model 3. Factors: (1) Eating Habits, (2) High Blood Glucose, (3) Thinness. χ^2^ = 99.7, df = 84, χ^2^/df = 1.19, CFI = 0.952, TLI = 0.940, RMSEA = 0.043, AIC = 171, ECVI = 1.68, ECVI LO-HI 90 = 1.53–1.97. (**d**) Model 4. Factors: (1) Eating Habits, (2) High Blood Glucose, (3) Thinness, (4) Avoidance. χ^2^ = 96.1, df = 81, χ^2^/df = 1.19, CFI = 0.954, TLI = 0.940, RMSEA = 0.043, AIC = 174, ECVI = 1.71, ECVI LO-HI 90 = 1.56–1.99. Note: Coefficients in curved lines indicate correlations. Coefficients in straight lines indicate standardized regression weights. The models are output of AMOS statistical software, where comma is used by default as a decimal separator. The numbers following commas indicate decimal points.

Comparison of one-, two-, three- and four-factor models of DEPS-R is shown in Figure 1. Goodness of Fit Indices (GFIs) suggested that all models fit the data well and their values were almost identical. Across any pair of compared models, the Delta chi square differences were largely insignificant (*p* > 0.40). The most preferable was model 1, because it is the most parsimonious while its GFIs are equal or even slightly better than those of competing models [23].

Reliability analysis suggested that the scale possessed an appropriate level of internal consistency (Cronbach’s alpha = 0.81).

### 3.3. Association between Patient Characteristics and Scores on DEPS-R

Patients with a score ≥20 were categorized as DEPS-R positive for ED. A threshold of 20 was selected based on previous studies that have demonstrated that a score of >20 indicates the need for further clinical evaluation of eating pathology [8,24,25]. Among the 103 participants, 31 (30.1%) scored above 20 points, the established DEPS-R cut-off score for disturbed eating behavior. Table 2 shows the comparison of characteristics between the DEPS-R positive and negative participants.

The DEPS-R positive group had higher values compared with the DEPS-R negative group of BMI (Median = 26.08 vs. 22.99) and HbA1 (Median = 7.0 vs. 6.5), and lower TIR levels (Median = 62.5 vs. 75.0). Weight (Median = 80 vs. 63) and TDD (Median = 46 vs. 35) were also significantly higher in the DEPS positive patients than in the DEPS negative patients.

In the final logistic regression model, as shown in Table 3, BMI and TIR remained as independent predictors for a positive DEPS-R screen. Specifically, for every point increase in BMI and TIR, the odds of being DEPS-R positive increased by 67% (OR = 1.33, 95% CI:1.10–1.60), or decreased by 4% (OR = 0.96, 95% CI:0.92–0.99), respectively.

## 4. Discussion

This is the first study to apply CFA on the results of the Greek version of the DEPS-R to investigate the construct validity of this measure, thus extending the validation provided earlier by application of exploratory factor analysis (EFA) [18] to the scores of a representative sample of insulin-treated adult patients with T1DM aged 19–72 years.

Through CFA, we focused primarily on comparison of the three-factor solution describing “Eating Habits”, “Thinness” and “High Blood Glucose” identified in previous research [9,10,16], with the single factor model originally proposed by Markowitz and colleagues (2010) [8]. We also considered two additional alternative models: (1) a four-factor model capturing the three aforementioned factors plus the “avoidance” factor that emerged in the Spanish study [14], (2) a two-factor model in which the “Eating Habits” and “Thinness” factors were combined in one factor, with “High Blood Glucose” as the second factor. Comparison of the models revealed that the one-factor model was the best among the four models for the Greek population of T1DM adult patients.

The methodological advance of the present study in comparison with the study conducted by Wisting and colleagues (2019) [10] is that all four models were nested, and included the same correlated residuals across all models. This allowed direct comparison between the models, which was not possible in Wisting’s non-nested models. The Goodness of Fit indices in the present study for all the nested models were similar to those of Wisting and colleagues (2019) when using a smaller number (three) of correlated residuals [10]. These findings support the structural validity of the DEPS-R in the Greek population of T1DM patients. More importantly, the superiority of the one-factor model for this population implies that Greek clinicians and practitioners will not need to consider individualized treatment based on various scores across different subscales (e.g., Eating Habits, Thinness, etc.), but they can adopt a single overall DEPS-R score ≥ 20 as suggested by DEPS-R inventors [8] for simple, effective screening for disordered eating in patients with T1DM.

In line with the previous study in Greece [18], the DEPS-R scale had acceptable internal consistency, suggesting that this tool is reliable for Greek T1DM patients. Our results also confirmed a significant positive association of DEPS-R score with BMI (≤0.001), TTD (*p* ≤ 0.001), HbA1c (*p* < 0.01) and TIR (*p* < 0.05), while it was also revealed that a higher BMI and TIR were independent predictors of a positive DEPS-R screen, implying more DE behaviors. In sum, all the aforementioned findings in this study are in line with previous studies supporting the convergent and external validity of the DEPS-R measure in Greek T1DM adult patients.

## 5. Conclusions

Early detection of disordered eating is critical for subsequent interventions to prevent T1DM patients’ serious diabetes complications. Alongside colleagues in other countries [8,9,10,11,12,13,14,15,16,17], Greek clinicians can trust the psychometric properties of the translated version of the DEPS-R and routinely use it for a rapid and easy screening of T1DM patients to identify those at risk of eating disorders. A single score ≥ 20 on DEPS-R [8] might be enough for the identification of Greek patients who are at risk of eating disorders. Nevertheless, to generalize this conclusion globally, the present methodology should be applied in future cross-cultural research.

## Figures and Tables

**Table 1:** Characteristics of patients with type 1 diabetes mellitus (T1DM) participating in the study (N = 103).

Characteristics	All (N = 103)	Male (N = 25)	Female (N = 78)	*p*-Value
Sociodemographic				
Age (Median, IQR; years)	37 (22)	40 (20.5)	35 (21.5)	0.035
Anthropometric				
Weight (Median, IQR; kg)	68.5 (22)	80 (13)	63 (15)	≤0.001
Height (Median, IQR; cm)	167 (14)	178 (13)	165 (9)	≤0.001
BMI (Median, IQR; kg/m^2^)	23.96 (5.79)	25.51 (3.94)	23.27 (5.83)	0.012
Clinical				
Diabetes Duration (Mean, SD; years)	18.32 ± 12.4	20.64 ± 12.38	17.58 ± 12.4	0.285 ^1^
Type of treatment (N; %)				
MDI	72 (69.9%)	15 (60%)	57 (73.1%)	0.322 ^2^
CSII	31 (30.1%)	10 (40%)	21 (26.9%)	
Use of CGM (N, %; Yes)	75 (73.5%)	20 (80%)	55 (71.4%)	0.560 ^2^
Total daily dose (Median, IQR; iu)	37 (18)	40 (23.5)	36.5 (20)	0.047
Glycemic control				
FBG (Mean, SD; mg/dL)	113.2 ± 31.42	115.32 ± 43.04)	112.53 ± 28.53	0.285 ^1^
HbA1c (Median, IQR; %)	6.8 (1)	6.8 (1)	6.8 (1)	0.754
BG measurements/day (Median, IQR)	7.0 (10)	5.5 (4.8)	7.0 (11)	0.010
Hypoglycemic events (Median, IQR)	5.0 (6)	5.0 (10)	5.0 (6)	0.844
TIR (Median, IQR; %)	71.5 (22.5)	79.5 (15.8)	68.5 (24.5)	0.058
TAR (Median, IQR; %)	19 (24)	14 (13)	20.5 (25)	0.144
TBR (Median, IQR; %)	6 (9)	8 (8)	5 (9)	0.440

Mean values and Standard Deviation (Mean, SD) are presented in normally distributed values. Median and interquartile range (Median, IQR) are presented in non-normally distributed values. Abbreviations: BMI: body mass index, MDI: multiple daily injections, CSII: continuous subcutaneous insulin infusion, CGM: continuous glucose monitoring, FBG: fasting blood glucose, HbA1c: Hemoglobin A1c, TIR: time in range (70–180 mg/dL); TBR: time below 70 mg/dL; TAR: time above 180 mg/dL. *P* value in bold if statistically significant at *p* ≤ 0.05.^1^ Independent samples *t*-test ^2^ Continuity correction applied.

**Table 2:** Characteristics of patients with type 1 diabetes mellitus (T1DM) according to the score on the Diabetes Eating Problem Survey-Revised Version (DEPS-R) (cut-off score 20) (N = 103).

Characteristics	DEPS-R Negative	DEPS-R Positive	*p*-Value
	(N = 72)	(N = 31)	
Sociodemographic			
Gender (N; %)			
Male	18 (25%)	7 (22.6%)	0.990 ^1^
Female	54 (75%)	24 (77.4%)	
Age (Median, IQR; years)	37 (23.8)	37 (21)	0.986
Anthropometric data			
Weight (Median, IQR; kg)	63 (19)	80 (20)	≤0.001
Height (Median, IQR; cm)	167 (13)	167 (13)	0.727
BMI (Median, IQR; kg/m^2^)	22.99 (5.05)	26.08 (8.46)	≤0.001
Clinical			
Diabetes Duration (Mean, SD; years)	17.92 (13)	19.26 (11.04)	0.617 ^2^
Type of treatment (N; %)			
MDI	50 (69.4%)	22 (71%)	1.000 ^1^
CSII	22 (30.6%)	9 (29%)	
Use of CGM (N, %)			
No	17 (23.6%)	10 (33.3%)	0.443 ^1^
Yes	55 (76.4%)	20 (66.7%)	
Total daily dose (Median, IQR; iu)	35 (15)	46 (31)	≤0.001
Glycemic control			
FBG (Mean, SD; mg/dL)	108.71 ± 26.86	121.85 ± 37.78	0.078 ^2^
HbA1c (Median, IQR; %)	6.5 (1)	7 (2)	0.004
BG measurements/day (Median, IQR)	7.0 (7.5)	7.0 (20)	0.295
Hypoglycemic events (Median, IQR)	5.5 (6.5)	5 (7.5)	0.16
TIR (Median, IQR; %)	75 (21.5)	62.5 (25)	0.027
TAR (Median, IQR; %)	16.5 (23)	20 (22)	0.202
TBR (Median, IQR; %)	6 (8)	5 (11)	0.94

Abbreviations—BMI: body mass index, MDI: multiple daily injections, CSII: continuous subcutaneous insulin infusion, CGM: continuous glucose monitoring, FBG: fasting blood glucose, HbA1c: Hemoglobin A1c, TIR: time in range (70–180 mg/dL), TBR: time below 70 mg/dL, TAR: time above 180 mg/DL. In bold if statistically significant at *p* ≤ 0.05. ^1^ Continuity correction applied. ^2^ Independent samples *t*-test.

**Table 3:** Multiple logistic regression analysis of factors associated with a high score on the Diabetes Eating Problem Survey-Revised Version (DEPS-R) of patients with type 1 diabetes mellitus (T1DM).

	OR (95% CI)	*p*-Value
BMI (kg/m^2^)	1.33 (1.10–1.60)	0.003
TIR (%)	0.96 (0.92–0.99)	0.022

BMI: body mass index, TIR: time in range (70–180 mg/dL). In bold if statistically significant at *p* ≤ 0.05. Weight was excluded as BMI is a function of weight.

## Data Availability

Data are available from the corresponding author upon reasonable request.
